# Effects of Internet Adoption on Health and Subjective Well-Being of the Internal Migrants in China

**DOI:** 10.3390/ijerph192114460

**Published:** 2022-11-04

**Authors:** Yihan Guo, Junling Xu, Yuan Zhou

**Affiliations:** 1 Department of Cultural Industries and Management, School of Media and Communication, Shanghai Jiao Tong University, No. 800 Dongchuan Road Minhang District, Shanghai 200422, China; 2 School of Public Administration, Central China Normal University, No. 152 Luoyu Road, Wuhan 430079, China

**Keywords:** internet adoption, rural–urban migrants, health status, subjective well-being, social capital

## Abstract

During the past decades, the number of rural–urban migrants has dramatically increased in China. Their well-being is important for social development and has attracted the attention of researchers. This paper adopts five waves of repeated cross-sectional datasets within a nine-year span, included in the Chinese General Social Survey (CGSS 2010–2018), to evaluate the impacts and mechanisms of internet adoption on the health status and subjective well-being of rural–urban migrants. Empirical results suggest that there are significant positive correlations between internet adoption and health status as well as subjective well-being. The results of structural equation modeling suggest that the impact of the internet on well-being occurs through increasing, bridging, and bonding social capital for rural–urban migrants. The mediating impact of bonding social capital on subjective well-being is more prominent, while the mediating impact of bridging social capital on health is stronger. Furthermore, we have explored the heterogeneous effects across gender and education. This is an early study which investigates such an important topic in the context of the digital era.

## 1. Introduction

Economic transformation during the past decades has led to rapid urbanization and accelerated rural–urban migration in China. In 2020, China’s population of domestic migrants reached 376 million, an increase of 69.73%, from 2010 (National Statistical Bureau). This population has migrated from rural to urban areas for better employment, salary, and quality of life [1,2]. They have, in the meantime, contributed to the rapid economic growth and social development of China. A large volume of literature has studied the causes and effects of their well-being. However, the hukou system, a household registered system, divides the residents’ identities and related public service and welfare systems into the rural and the urban, strengthening the rural–urban gap and its barriers [3]. Thus, the migrants cannot access equal public resources enjoyed by urban residents, and their social integration and urbanization are consequently hindered. It is widely documented that migrants experience lower levels of well-being than local citizens due to their difficulties in adapting to city living or expectations of success [4].

The world is facing a crucial and dramatic transition: social and economic challenges related to the advancement of digital technologies. The global internet penetration rate now stands at 59.5%, and around 4.66 billion people worldwide use the internet [5]. As of December 2021, the number of internet users in China reached 1.01 billion, accounting for 71.6% of the total population. Online social engagement has become an important part of citizens’ lives, and most of the economic and social activities have moved online during the past decade. Does online engagement help the social integration of rural–urban migrants to improve their health status and subjective well-being?

To date, a large body of literature has found positive effects of internet use, including reducing loneliness [6,7,8] and a higher level of self-reported satisfaction as well as a lower chance of being isolated [9,10]. Xu and Huang used cross-sectional data and found that internet use enhances happiness by reducing loneliness and increasing volunteering [11]. However, the existing literature has barely examined the roles of internet technologies in the well-being of rural–urban migrants, and never considers its mechanism. Our study extends this branch of literature, examining the impacts of internet adoption on the health condition and subjective well-being (SWB) of migrants.

Additionally, existing studies have found clear gender segregation and division of labor among Chinese migrants [12]. Meanwhile, the proportion of highly educated migrants presents a rising tendency in China (National Statistical Bureau, Beijing, China), and a positive correlation between well-being and education among general individuals is revealed [13]. Does internet use impact each user according to their individual characteristics, such as gender and education?

Empirically, this study uses the 2010, 2013, 2015, 2017, and 2018 data of the Chinese General Social Survey (CGSS) to evaluate the impacts of internet adoption on the health status and subjective well-being of rural–urban migrants. Importantly, structural equation modeling (SEM) is used to estimate the relationships and the mechanisms. Furthermore, we explored the heterogenous effects of gender and education.

## 2. Theoretical Background

### 2.1. SWB of the Rural–Urban Migrants

Subjective well-being is an important indicator of individual and social welfare and reveals the overall evaluation of the living qualities one experiences. The SWB of the rural–urban migrants has also attracted the wide attention of researchers and policymakers. As discussed in the introduction section, rural–urban migrants reported lower levels of well-being than local citizens or even lower within a self before–after comparison survey. Aside from the traditional factors in the function of SWB (such as, for example, common personal characteristics and socioeconomic factors), institution concerns, social structure, family arrangement, and social integration are also widely taken into consideration in the current research of Chinese migrants [4,14,15,16,17].

Social support and integration play an important role in closing the well-being gap between immigrants and local city residents [18]. Strong social ties directly increase the SWB of migrants [19]. Theoretically, social integration is the process of the convergence or transformation of migrants to locals in terms of economy, psychology, culture, and identity. In this process, individuals with a low degree of integration are often more susceptible to external discrimination, which in turn leads to a decline in well-being [20,21]. Existing research has suggested that social media use can contribute to urban migrants’ social integration [22,23].

### 2.2. Health Status of the Rural–Urban Migrants

Health status is one important factor in the function of SWB and has also attracted wide attention. It is also an important form of human capital, influencing productivity. Determinants of mental and physical health for the rural–urban migrant commonly identified in the existing literature include but are not limited to age, gender, education, marital status, social status, personal income, household wealth, family structure, living arrangement, social network, social integration, and working environment [14,24,25,26,27]. Migrants, although moving for economic opportunities, are not guaranteed to experience better health status [26]. Generally, individuals with different races, education levels, and different socioeconomic characteristics show different health statuses [28,29,30]. Improved social networks and social integration have also generated significant impacts on the health statuses of rural–urban migrants. Social support and migrants’ confidence are two important mechanisms in the above positive relationships [14,27,31]. However, internet technological factors have been overlooked in the literature on the health of migrants, and this study contributes to the literature from this perspective.

### 2.3. Internet, Social Capital, and Well-Being of the Rural–Urban Migrants

Since the rapid development of internet technology in the last two decades, studies on the relationship between internet use and subjective well-being have been increasing. However, a consensus on the effects of internet use on the well-being of individuals has not yet been reached. The use of the internet could be conceived as part of a set of diverse contributory antecedents and functions related to well-being [32]. Some studies have found that, the more time one spends on the internet and on using instant messages, the more likely they are to be associated with high levels of depression, loneliness, and a decrease in happiness [33,34,35]. Another branch of literature supported the positive impacts of the internet on individuals’ subjective well-being. When the internet is used to give and receive support, particularly by increasing social contact among close relations, it enhances well-being [36].

Social capital is a complex and multidimensional concept encompassing trust, institutions, social networks, and cultural and social value systems [37,38,39]. Especially, social networks are considered one of the most central domains, reflecting how people link with each other, while social capital is created in the networks [40,41]. Granovetter [42] divided social networks into strong ties and weak ties, and Putnam further defined the attributes of weak ties and strong ties as bridging and bonding social capital. While bonding social capital refers to intra-group ties between members sharing similar demographic characteristics, bridging social capital refers to the connection between heterogeneous individuals [43,44]. Strong ties, such as family, are related to bonding social capital in that they provide emotional and social support. Meanwhile, weak ties, including acquaintances and colleagues, are associated with bridging social capital; hence, they are more likely to provide new information [45]. Previous studies have hypothesized four paths or mechanisms by which social capital may affect health and behavior: informational support, instrumental support, emotional support [46], and collective efficacy [47,48]. The existing literature has provided evidence that participation in social networks and social activities is beneficial for an individual’s subjective well-being [49,50,51]. The main channels of the network effect are through strengthening social cohesion and social participation to increase migrants’ confidence and to reduce their anxiety [14,52]. It can thereby narrow the happiness gap between the migrants and local city residents.

Internet use could complement face-to-face social interaction and provide ways of online communication and information [53,54,55]. In this way, social connections and social support are enhanced and improved (the so-called capitalization effect [56]). Social network construction through internet use is a form of social capital which strengthens the sense of identity and promotes social integration. Therefore, it is likely that the use of this internet technology can enhance subjective well-being through social capital cultivation [36,57,58].

However, in existing studies about migrants, the mediating effects of bridging social capital and bonding social capital in the relationships between internet use and well-being have barely been studied empirically; moreover, hardly any have focused on the group of rural–urban migrants in China. Social integration is one of the main obstacles to their lives in urban areas. Our paper builds and extends this branch of literature on the impacts of internet technology adoption on the health and well-being of rural–urban migrants in China. Moreover, the mechanisms of social capital cultivation are also investigated.

**Figure 1:**
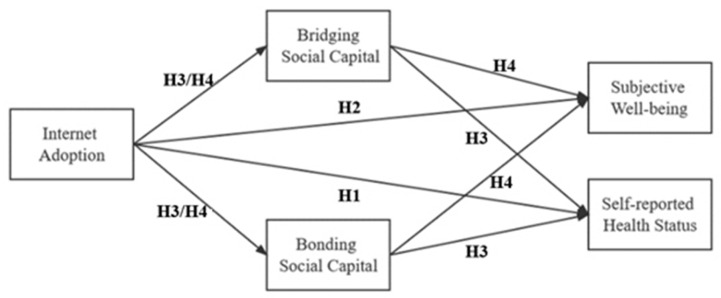
Conceptual framework.

Based on the above discussion, and combined with the research objectives, we put forward our hypotheses in this paper: internet technology adoption influences the health status and subjective well-being of rural–urban migrants, and social capital plays a mediating role in the process of this influence. Specifically, the following assumptions are included and summarized in Figure 1:

**Hypothesis** **1.** *Internet adoption affects the health status of rural–urban migrants. The higher the frequency of internet adoption, the better one’s health.*

**Hypothesis** **2.** *Internet adoption affects the subjective well-being of rural–urban migrants. The higher the frequency of internet adoption, the better the subjective well-being.*

**Hypothesis** **3.** *Social capital has a positive mediating effect on the mechanism of the impact of internet adoption on the health status of rural–urban migrants.*

**Hypothesis** **4.** *Social capital has a positive mediating effect on the mechanism of the influence of internet adoption on the subjective well-being of rural–urban migrants.*

## 3. Research Design

### 3.1. Data Sources

The main research samples in this study came from the five rounds of the Chinese General Social Survey (CGSS) from 2010 to 2018, a nationwide sampling and repeated survey project initiated by the National Survey Research Center at the Renmin University of China. It provides detailed and scientific basic information for social science research and government decision-making. The survey area covered 31 provinces (autonomous regions and municipalities directly under the central government) across the country, and 7000 to over 10,000 families were visited in each survey.

We kept the respondents whose hukou location was different from their current living place. After this, 6212 observations were left, and the yearly sample distribution is presented in Table 1. The scale of age was concentrated from 18 to 92, with an average age of about 42. Women accounted for 50.26%, slightly higher than men. The samples with agricultural hukou accounted for 53.51%, slightly lower than that of the 54.29% for the total population. Moreover, 73.66% of the respondents were in a long-term relationship or marriage. In education, 18.24% of the respondents received primary education or below, 50.00% secondary education or the same level of education, and 31.76% university education or above. In income, 14.33% of the respondents had no income, 35.99% had an annual income of 30,000 yuan or less, 21.15% had an income of 30,001 to 50,000 yuan, 19.39% had an income of 50,000 to 100,000 yuan, and 9.14% had an annual income of more than 100,000 yuan. In the subjective perception of social class, over 90% of the samples considered themselves as middle class and beyond, and only 7.49% of them were in the relatively high or highest class. Descriptive statistics are shown in Table 2.

### 3.2. Measurements

Internet adoption (IA) was measured through the respondents’ responses to the question “How often have you used the internet in the past year?” ranging from 1 to 5, representing “never” to “very frequently”. A higher score implies a higher level of IA (M = 3.44, SD = 1.62).

Subjective well-being (SWB) was measured through the respondents’ responses to the question “In general, how happy do you think your life is?” ranging from 1 to 5, representing “very unhappy”, “relatively unhappy”, “so so”, “relatively happy”, and “very happy”, respectively. A higher score indicates a higher level of SWB (M = 3.87, SD = 0.80).

Self-reported health status (SRHS) was measured through the respondents’ responses to the question “How do you feel about your current physical health?”, also ranged from 1 to 5, representing “very unhealthy”, “relatively unhealthy”, “neutral”, “relatively healthy”, and “very healthy”, respectively. A higher score suggests a higher level of SRHS (M = 3.88, SD = 0.97). Additionally, the adoption of subjective self-reported indicators to effectively measure SRHS and SWB has been supported by existing studies and was verified to produce good reliability and validity [59,60,61,62,63].

For bridging social capital (BRSC) and bonding social capital (BOSC), considering the availability of data, the answer to “the frequency of meeting with relatives who do not live together in free time” was taken as the proxy variable of BOSC, and the answer to “the frequency of meeting with friends in free time” was taken as the proxy variable of BRSC. Two studies based on the CGSS and related series projects had adopted a similar method to measure social network strengths as an important part of social capital [64,65]. The bridging and bonding social capitals were also measured through answers ranging from 1 to 5. A higher score indicates a higher level of BRSC (M = 2.55, SD = 0.93)/BOSC (M = 2.21, SD = 0.73).

### 3.3. Analysis

The correlations between important variables were first reported, and then a structural model with two mediating variables was used to test the determinants of SWB and SRHS. Our studied variables (SRHS, SWB, IA, BRSC, and BOSC) were all measured through a five-point Likert scale; hence, we adopted Pearson correlations to estimate the correlations among them, supported by existing research [66,67]. The mediating variable facilitates an understanding of the effect path of the independent variable on the dependent variable [68]. We adopted Baron and Kenny’s [69] use of the casual step approach—the most popular method—to test the mediation model. In a simple mediating effect model, the casual step approach requires an estimation of the coefficient on each path from the indicator to the outcome, and then the confirmation of the mediating effect by judging whether the related statistical criteria are met. We also took advantage of the test process suggested by Wen and Ye [70], supported by recent studies [71,72,73]. The demographic and socio-economic characteristics of the respondents, including gender, age, marital status, education, income, subjective social class, as well as the fixed effect of province and time, were controlled to enhance the robustness of the results. Additionally, a 5000 times bootstrap method based on the “sgmediation” command of Stata developed by the University of California, Los Angeles (UCLA) was adopted to conduct Sobel–Goodman mediation tests as a robustness test.

Further, two heterogenous tests were conducted. We divided the sample into different subgroups according to their gender and education level to examine the heterogeneity of the effects. In this section, we divided the samples into different groups by education levels: uneducated, primary school or below, junior middle school, senior high school, and university or above. All of the data analysis was performed by Stata 16.0.

## 4. Results

### 4.1. Descriptive Statistics

The descriptive statistics of the variables and bivariate correlations are presented in Table 3. There are significant positive correlations among IA, SWB, and SRHS. The mediators, BRSC and BOSC, are also found to be significantly positively correlated with IA, SWB, and SRHS, respectively.

### 4.2. Structural Equation Modeling with Two Mediators

**Figure 2:**
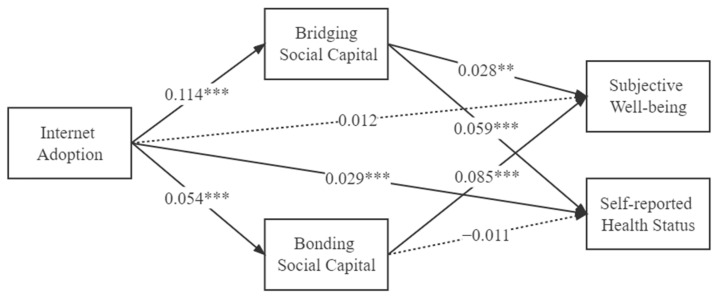
Results of structural equation modeling. Note: *** *p* < 0.01, ** *p* < 0.05.

The casual step approach is adopted to examine the mediating model. All the regressions control for socio-economic and demographic characteristics, survey year, and province-fixed effects widely considered in the existing literature [24,25,26,27]. The most important results are presented in Figure 2.

All path coefficients, with the only exception being the path BOSC mediating the effect of IA on SRHS, were in line with predictions. At first, the total effects of IA on SWB and SRHS are positive and significant at the 5% and 1% significance levels, respectively, before adding mediating variables. This indicates that, without considering channel variables, more frequent internet use is associated with higher SWB and SRHS levels among rural–urban migrants. The larger coefficient and higher significance level of IA affecting SRHS suggest that internet technological factors have exerted a greater impact on the SRHS of rural–urban migrants.

For mediating effects, in general, the results of the casual step approach suggest that IA could indirectly influence the SWB in rural–urban migrants by affecting BRSC and BOSC and impact on the SRHS in the rural–urban migrants by affecting BRSC.

In the case of SWB, the direct effects of IA on BRSC (β = 0.114, *p* < 0.01) and BRSC on SWB (β = 0.028, *p* < 0.05) are both positive and significant. This confirms a positive correlation between the IA and BRSC of Chinese rural–urban migrants, and an increase in each unit of BRSC could lead to an increased SWB by 0.028. Hence, the indirect path from IA via BRSC to SWB is significant, with an effect size of 0.003, accounting for 16.0% of the total effect. Meanwhile, we also find that the direct effects of IA on BOSC (β = 0.054, *p* < 0.01) and BOSC on SWB (β = 0.085, *p* < 0.01) are positive and significant as well. This demonstrates that the accumulation of BOSC and its strong ties through the internet leads to a higher level of SWB for Chinese rural–urban migrants, with an effect size of 0.005, compared with BRSC and weak ties; moreover, the indirect effect size on SWB of the latter is only about 32.9% of the former. Finally, after adding BRSC and BOSC into the regressions, the effect of IA on SWB turns insignificant, demonstrating the fully mediating role that BRSC and BOSC played in this channel. This suggests that the positive effect of the internet on the SWB of Chinese rural–urban migrants is largely based on an increase in social capital.

In the case of SRHS, the direct effects of BRSC on SRHS (β = 0.059, *p* < 0.01) are positive and significant as well, suggesting that an increase in each unit of BRSC could lead to an increased in SRHS by 0.059. Therefore, combined with the significant effect of IA on BRSC mentioned in the previous section, the mediating channel from IA via BRSC to SWB is significant, with an effect size of 0.007, accounting for 19.2% of the total effect. However, the mediating effect of BOSC did not pass the test since the effect of BOSC on SRHS was not significant. Following the test procedure recommended by Wen and Ye [70], a 5000 times bootstrapping was used to retest the mediating effect of BOSC, and its indirect effect is still insignificant (β = 0.001, *p* = 0.414). Thus, in general, the effect of the internet on health through social capital channels accounts for only a part of the total effect. Finally, after adding BRSC and BOSC into the regressions, the effect of IA on SRHS remains significant, revealing the partially mediating role BRSC played in this channel.

Moreover, estimations using bootstrap samplings as a robustness test show similar results. All results are summarized in Table 4.

Additionally, we also find that an older age is associated with a lower level of self-reported health status, with a rate of −0.017 per year. Nevertheless, the SWB rate slightly increases by 0.3% with each year of age. Owning a rural hukou is associated with a higher SRHS by 13.1%. The coefficients of perceived social class are generally large, especially for SWB, and all are significant, at the 1% significance level. Income only has a significant positive effect on SRHS. Being in a long-term relationship or marriage has a significant positive effect on SWB; however, its impact on SRHS is barely significant, with a much smaller coefficient. Education has an insignificant effect on both SHRS and SWB. For most of the individual-level variables, the coefficients and significance levels of their effects on SWB or SRHS show little change after controlling for social capitals, suggesting that social capital channels play a small role. As for the effects of the variables on the two social capitals, the magnitude BRSC affected by external factors is intuitively larger than BOSC, which is consistent with the characteristics of BRSC and weak ties declared by Putnam [43] and Granovetter [42]. Detailed results including control variables are shown in Appendix A (Table A1).

### 4.3. Heterogeneous Effect

#### 4.3.1. Heterogeneous Effects of Gender

Table 5 reports the heterogeneous effects of different gender on migrants’ SWB and SRHS. In general, we find interesting differences in the effect of IA on the two dependent variables between male and female migrants. For females, IA could positively impact the SWB of female migrants by enhancing both social capitals; however, its effect on SRHS through both social capital channels is not supported. For males, the mediation of BRSC in the effects of IA on SRHS is supported, while the mediation of ORSC in the effects of IA on SWB is supported.

In terms of the female subsample, the internet could influence their SWB by affecting BRSC and BOSC, with an approximate effect size of 0.004, while the mediators play full mediation roles. However, neither channel effect of the two social capitals on SRHS is significant since the social capitals could not directly impact the SRHS of female respondents. Moreover, the significance level and coefficient of the bonding social capital are both higher than bridging social capital. In addition, the effect of education level on SWB is significant, though is found to be nonsignificant in the full sample.

In terms of the male subsample, the internet could impact their SWB through affecting BOSC, with an effect size of 0.005, which is higher than that of 0.004 in females. Meanwhile, the internet could influence their SRHS through the channel of BRSC, with an effect size of 0.009. Apparently, the two channels of social capital play distinctive roles, respectively.

#### 4.3.2. Heterogeneous Effects of Education Level

Table 6 reports the heterogeneous effects of different education levels. In general, for SWB, the effect of the BOSC channel is relatively prominent. For SRHS, the effect of the BOSC channel is not stably significant compared with BRSC.

First, in the uneducated group, IA fails to influence the two outcome variables directly or indirectly. It only enhances the samples’ BRSC significantly in a large magnitude. Second, in the primary school group, the internet also fails to produce the total and direct effects on SWB and SRHS. However, through the indirect effecting channel of BOSC, the internet could influence SWB at a 0.008 effect size. Moreover, through the influencing channel of BRSC, it could impact SRHS at an effect size level of 0.012. These are both full meditations. Third, in the junior middle school group, the total effect of IA on SWB is highly significant, and all indirect channels are significant except IA via BRSC on SWB. As with the former groups with lower education, for the junior high school-educated individuals, their happiness through the internet may mostly come from strong ties. However, the coefficient of BOSC to SRHS is negative, suggesting that the strong ties might have a negative effect on their SRHS. Fourth, in the senior high school group, IA can directly impact SRHS, but only the mediating channel of BOSC influences SRHS as a partial mediator. Moreover, the positive effects of IA on SWB through BOSC and BRSC channels are both significant and fully mediated. Fifth, in the university or above group, the total and direct effects of the internet are both nonsignificant, and only the mediating channels of BRSC is significant with a 0.005 effect size. Moreover, the r-square in this group is lower than that in the other groups, indicating that the internet plays a smaller role in the SWB of the highly educated rural–urban migrants.

## 5. Discussion

This study focused on the rural–urban migrant group in China and explored the impact of information technology’s adoption on their physical and mental health and its mechanisms with the social capital theory proposed by Putnam [43]. A mediation model analysis based on the casual step approach is conducted.

First, the results of the total effects support the previous conclusion of a positive impact of the internet on SWB [36] and notably reveal a positive correlation between internet use and health status, which was almost ignored in the literature, in Chinese rural–urban migrants.

Second, the mediation effect test shows that, for Chinese rural–urban migrants, internet adoption could positively affect their subjective well-being directly, as well as by enhancing bridging and bonding social capital. Moreover, the effect of bonding social capital was larger than that of bridging social capital. It can be explained that China has a strong family tradition, and kinship ties and clan concepts exert a profound effect on Chinese culture, especially for the rural population, which accounts for more than half of the samples. The cross-regional function of the internet may enable migrants to interact more with those who have strong ties with them such as relatives, thereby improving their well-being. Although the internet has promoted individuals to access more BRSC as well, the magnitude of its improvement on Chinese migrants’ happiness is still lower than that of bonding social capital, especially family.

Third, only the partial mediation of bridging social capital in effect of internet on self-reported health is supported, and the effect of the internet on health through social capital channels accounts for only a part of the total effect. On one hand, the internet adoption of Chinese rural–urban migrants has brought more weak ties, whose benefit exchange function is more conducive to the transfer of social resources in aspects such as health information and medical resources, thereby improving their health. On the other hand, the help provided by the internet’s information function itself, such as knowing one’s health status and acquiring information on medical resources, may be obtained without relying on social relations to a greater extent for the migrants. Meanwhile, after adding social capitals into the regressions, the effect of the internet on health remains significant, revealing the partially mediating role that bridging social capital played in this channel. The r-square also changes little, revealing that it may exist in more channels to potentially explore in the future.

Additionally, heterogeneous effects across gender and education are explored and show interesting differences in the effect internet adoption has on the two dependent variables between male and female migrants. For females, internet adoption could positively impact the SWB of female migrants by enhancing both social capitals; however, the mediations are found to be nonsignificant in the paths on health. Social interaction on the internet may be more about emotional support for female rural–urban migrants, compared with the exchange of interests. At this point, enhancing the strong ties of female migrants may improve their mental health more effectively. For males, the two social capital channels play distinctive roles, respectively, in the effect of internet adoption on the two dependent variables. The mediation of bridging social capital in the effects of internet adoption on self-reported health is supported, while the mediation of bonding social capital in the effects of internet adoption on SWB is supported. The internet improves their bridging social capital and weak ties such as ordinary friends and acquaintances, which may help men more efficiently organize health activities and share health status or information. Meanwhile, to a greater extent, their true happiness still comes from their families and clans. In the traditional social concept of East Asia, men are generally regarded as the “pillar” of the family and the “heirs” of surnames. They are given greater attention within the family and are more closely related to their families. Meanwhile, the heterogenous test of education reveals little tendency. For well-being, the effect through the bonding social capital channel is relatively prominent. For self-reported health, the effect of the bonding social capital channel is not stably significant compared with bridging social capital. A finding worth noting is that neither direct nor indirect channels of internet on dependent variables are significant. The average age of the uneducated group is 58, and 78% of the samples are 45 years old or above. Obviously, they are mostly middle-aged and elderly, and the enabling function of internet may be limited due to their education levels.

This study can contribute to the literature. First, although there is a large body of literature on the effects of internet use on physical and mental health, few studies have explored the effects and mechanisms of internal migrants. This study extends this topic into migrant research. Secondly, the study further explores the mediation of bridging and bonding social capital and the effect of the internet on health from the perspective of social capital. This is an early study of such an important topic in the context of the digital era. It is found that both social capitals could be mediators of the internet’s impacts on SWB, but that only bridging social capital mediates impacts on health. In this case, this study enriches current knowledge about migrants’ health and social integration.

This study has policy implications as well. In China, due to the rural–urban dual structure, internal migrants find difficulties in adapting to city living or the expectations of success, facing higher survival pressure. This study reveals that the penetration and development of the internet may provide possible approaches to improve their situation. On the one hand, to better capitalize on the role of the internet in promoting their health and well-being, measures can be adopted to help rural–urban migrants improve in their digital literacy and mitigate the rural–urban digital divide. On the other hand, migrants could be encouraged to develop online and offline health and social activities to help them expand social networks, build social capital, and improve their health and social integration. Meanwhile, targeted measures can be applied to gender-specific and illiterate groups.

Finally, we would like to mention some pitfalls of this study. On the one hand, this paper chose Chinese residents as the research object; thus, the universality of the results needs to be improved. On the other hand, limited by the conditions of the dataset, the two social capitals in this paper were measured using single-survey items as the proxy variables, which only reflect part of the social capital in the aspect of social network strength [64,65]. However, future studies should consider using multiple survey items to describe more dimensions of social capital, a complex and diverse concept.

## 6. Conclusions

This study extends the discussion of the effects of the internet on subjective well-being and self-reported health to the field of migrant research and explores the mediation channels based on social capital theory. A mediation model analysis based on the casual step approach supports the mediation of both the bridging and bonding of social capital in the impact of the internet on SWB, as well as the mediation of bridging social capital in the impact on health. Further, the result of heterogeneous effect analysis found that there were distinct differences in the influences between different gender and education groups. In the process of economic transformation and urbanization, our findings broaden our understanding of the effects produced by the internet in improving migrants’ physical and mental health situations.

## Figures and Tables

**Table 1:** Annual distribution of the sample.

Year of Survey	Respondents	Percentage
2010	931	14.99%
2013	973	15.66%
2015	1020	16.42%
2017	1645	26.48%
2018	1643	26.45%

**Table 2:** Descriptive statistics of sampling characteristics (*n* = 6212).

Variables	Freq.	Percentage
Female		
* Mean (SD)*		0.50 (0.50)
Age		
* Mean (SD)*		41.83 (16.01)
Subjective social class		
* Mean (SD)*		2.47 (0.84)
* =1 lowest class*	885	14.25%
* =2 low class*	2040	32.84%
* =3 middle class*	2822	45.43%
* =4 high class*	426	6.86%
* =5 highest class*	39	0.63%
Education		
* Mean (SD)*		3.63 (1.20)
* =1 Illiterate*	306	4.93%
* =2 Primary school*	827	13.31%
* =3 Junior high school*	1700	27.37%
* =4 Senior high school*	1406	22.63%
* =5 College and above*	1973	31.76%
Rural Hukou		
* Mean (SD)*		0.54 (0.50)
Marriage		
* Mean (SD)*		0.74 (0.44)
Income (ln)		
* Mean (SD)*		9.01 (3.82)

**Table 3:** Descriptive analysis and correlational coefficients of studied variables.

	Mean	SD	1	2	3	4	5
1. Subjective Well-being	3.87	0.80	1.00				
2. Self-reported Health Status	3.88	0.97	0.18 ***	1.00			
3. Internet Adoption	3.46	1.62	0.03 **	0.22 ***	1.00		
4. Bridging Social Capital	2.55	0.93	0.05 ***	0.16 ***	0.33 ***	1.00	
5. Bonding Social Capital	2.21	0.73	0.11 ***	0.04 ***	0.13 ***	0.39 ***	1.00

Note: *** *p* < 0.01, ** *p* < 0.05.

**Table 4:** Coefficients of structural equation modeling.

Path	Coef.	Robust SE	*p*-Value
Total effect			
Internet Adoption→Subjective Well-being	0.020	0.009	0.033
Internet Adoption→Self-reported health status	0.035	0.011	0.001
Direct effect			
Internet Adoption→Subjective Well-being	0.012	0.009	0.190
Internet Adoption→Self-reported health status	0.029	0.011	0.008
Internet Adoption→Bridging Social Capital	0.114	0.010	0.000
Internet Adoption→Bonding Social Capital	0.054	0.009	0.000
Bridging Social Capital→Subjective Well-being	0.028	0.013	0.036
Bonding Social Capital→Subjective Well-being	0.085	0.015	0.000
Bridging Social Capital→Self-reported health status	0.059	0.015	0.000
Bonding Social Capital→Self-reported health status	−0.011	0.018	0.542
Indirect effect	Effect size	Bias-corrected LLCI	Bias-corrected ULCI
Internet Adoption→Bridging Social Capital→Subjective Well-being	0.003	0.000	0.005
Internet Adoption→Bonding Social Capital→Subjective Well-being	0.005	0.001	0.004
Internet Adoption→Bridging Social Capital→Self-reported health status	0.007	0.003	0.009
Internet Adoption→Bonding Social Capital→Self-reported health status	-	-	-

Note: The LLCI and ULCI of indirect effects were calculated by bootstrap method, and the rest used the casual step approach.

**Table 5:** Heterogeneous effect of gender.

	(1)	(2)	(3)	(4)	(5)	(6)
Outcome	SWB	SRHS	BRSC	BOSC	SWB	SRHS
Female group						
IA	0.019	0.039 **	0.123 ***	0.057 ***	0.011	0.035 **
	(1.40)	(2.43)	(8.15)	(4.52)	(0.79)	(2.13)
BRSC					0.035 *	0.034
					(1.96)	(1.53)
BOSC					0.069 ***	0.007
					(3.12)	(0.26)
Observations	3122	3122	3122	3122	3122	3122
R-squared	0.098	0.160	0.186	0.062	0.104	0.161
Male group						
IA	0.022 *	0.032 **	0.104 ***	0.050 ***	0.015	0.024
	(1.69)	(2.11)	(7.20)	(4.04)	(1.17)	(1.59)
BRSC					0.017	0.085 ***
					(0.87)	(3.93)
BOSC					0.103 ***	−0.027
					(4.75)	(−1.08)
Observations	3090	3090	3090	3090	3090	3090
R-squared	0.101	0.156	0.170	0.056	0.111	0.161

Note: Year- and province-fixed effects are both controlled. Robust t-statistics in parentheses. *** *p* < 0.01, ** *p* < 0.05, * *p* < 0.1. IA = Internet Adoption; BRSC = Bridging Social Capital; BOSC = Bonding Social Capital; SWB = Subjective Well-being; SRHS = Self-reported Health Status.

**Table 6:** Heterogeneous effect of education.

	(1)	(2)	(3)	(4)	(5)	(6)
Outcomes	SWB	SRHS	BRSC	BOSC	SWB	SRHS
Illiterate group						
IA	−0.019	−0.011	0.151 **	0.077	−0.025	−0.026
	(−0.34)	(−0.18)	(2.13)	(1.57)	(−0.44)	(−0.40)
BRSC					−0.032	−0.044
					(−0.60)	(−0.62)
BOSC					0.137 *	0.274 ***
					(1.80)	(2.60)
Observations	306	306	306	306	306	306
R-squared	0.245	0.203	0.242	0.115	0.254	0.227
Primary Education group						
IA	0.021	0.031	0.144 ***	0.080 ***	0.016	0.025
	(0.86)	(0.98)	(4.52)	(3.34)	(0.66)	(0.79)
BRSC					−0.024	0.083 **
					(−0.80)	(2.08)
BOSC					0.102 ***	−0.078
					(2.60)	(−1.50)
Observations	827	827	827	827	827	827
R-squared	0.167	0.168	0.104	0.096	0.173	0.174
Junior high school Education group						
IA	0.041 ***	0.021	0.115 ***	0.054 ***	0.034 **	0.016
	(2.61)	(1.16)	(6.47)	(3.65)	(2.21)	(0.89)
BRSC					0.020	0.081 ***
					(0.83)	(2.81)
BOSC					0.072 **	−0.087 **
					(2.43)	(−2.45)
Observations	1700	1700	1700	1700	1700	1700
R-squared	0.128	0.182	0.127	0.051	0.134	0.188
Senior high school Education group						
IA	0.000	0.050 **	0.119 ***	0.046 **	−0.013	0.041 *
	(0.02)	(2.19)	(5.67)	(2.51)	(−0.66)	(1.80)
BRSC					0.059 *	0.045
					(1.87)	(1.44)
BOSC					0.135 ***	0.063 *
					(3.95)	(1.77)
Observations	1406	1406	1406	1406	1406	1406
R-squared	0.091	0.209	0.176	0.044	0.111	0.214
College degree and above group						
IA	0.017	0.023	0.065 ***	0.023	0.014	0.019
	(0.77)	(0.86)	(2.72)	(1.01)	(0.60)	(0.69)
BRSC					0.039	0.074 **
					(1.56)	(2.51)
BOSC					0.052 **	−0.007
					(1.99)	(−0.23)
Observations	1973	1973	1973	1973	1973	1973
R-squared	0.098	0.101	0.092	0.036	0.103	0.105

Note: Year- and province-fixed effects are both controlled. Robust t-statistics in parentheses. *** *p* < 0.01, ** *p* < 0.05, * *p* < 0.1.

## Data Availability

We promise that the data is available when required and permission to reproduce material from other sources.

## Appendix A

**Table A1:** The effect of IA on SWB and SRHS and the mediating role of two social capitals.

	(1)	(2)	(3)	(4)	(5)	(6)
Outcome	SWB	SRHS	BRSC	BOSC	SWB	SRHS
IA	0.020 **	0.035 ***	0.114 ***	0.054 ***	0.012	0.029 ***
	(2.14)	(3.22)	(10.92)	(6.14)	(1.31)	(2.64)
BRSC					0.028 **	0.059 ***
					(2.10)	(3.80)
BOSC					0.085 ***	−0.011
					(5.55)	(−0.61)
Perceived social status	0.207 ***	0.139 ***	0.069 ***	0.048 ***	0.201 ***	0.135 ***
	(15.49)	(9.61)	(5.07)	(4.17)	(15.06)	(9.37)
Age	0.003 ***	−0.017 ***	−0.006 ***	0.001	0.003 ***	−0.017 ***
	(3.76)	(−15.96)	(−6.13)	(1.47)	(3.86)	(−15.53)
Education	0.017	0.005	0.055 ***	0.041 ***	0.012	0.002
	(1.44)	(0.38)	(4.17)	(3.78)	(1.02)	(0.17)
lnincome	−0.004	0.012 ***	0.013 ***	0.008 ***	−0.005	0.011 ***
	(−1.27)	(3.37)	(4.06)	(2.92)	(−1.63)	(3.19)
Marriage	0.166 ***	0.051 *	−0.202 ***	0.061 ***	0.166 ***	0.063 **
	(6.59)	(1.82)	(−7.45)	(2.76)	(6.60)	(2.26)
Female	0.062 ***	−0.109 ***	−0.100 ***	0.040 **	0.061 ***	−0.103 ***
	(3.05)	(−4.64)	(−4.53)	(2.10)	(3.03)	(−4.36)
Rural Hukou	−0.033	0.131 ***	−0.124 ***	−0.061 ***	−0.024	0.137 ***
	(−1.34)	(4.69)	(−4.66)	(−2.74)	(−1.00)	(4.91)
Year of survey fixed effect	Yes	Yes	Yes	Yes	Yes	Yes
Province-fixed effect	Yes	Yes	Yes	Yes	Yes	Yes
Constant	2.963 ***	4.016 ***	2.125 ***	1.611 ***	2.767 ***	3.909 ***
	(30.73)	(37.00)	(20.34)	(18.29)	(27.56)	(34.21)
Observations	6212	6212	6212	6212	6212	6212
R-squared	0.095	0.156	0.174	0.050	0.103	0.159

Note: Robust t-statistics in parentheses. *** *p* < 0.01, ** *p* < 0.05, * *p* < 0.1.
